# Analysis of the treatment of RT2 recessions with a xenogeneic collagen matrix vs. connective tissue graft combined with a coronally advanced flap. A double-blinded randomized clinical trial

**DOI:** 10.1007/s00784-024-05602-9

**Published:** 2024-03-15

**Authors:** Ruiz-de-Gopegui-Palacios Elena, Vilor-Fernández Miren, García-De-La-Fuente Ana-María, Marichalar-Mendía Xabier, Aguirre-Zorzano Luis-Antonio

**Affiliations:** 1 University of the Basque Country (UPV/EHU), Biscay, Spain; 2 Research Group: GIU21/042. Department of Stomatology, Faculty of Medicine and Nursing, University of the Basque Country (UPV/EHU), Barrio Sarriena s/n, Biscay, Leioa 48940 Spain; 3 Research Group: GIU21/042. Department of Nursery I. Faculty of Medicine and Nursing, University of the Basque Country (UPV/EHU), Biscay, Spain; 4 Research Group: GIU21/042. Department of Stomatology, University of the Basque Country (UPV/EHU), Biscay, Spain

**Keywords:** Collagen matrix, Gingival recession, Connective tissue, Plastic surgery procedures, Clinical trial

## Abstract

**Objectives:**

To compare the clinical efficacy in terms of mean root coverage in RT2 recession treated with a coronally advanced flap combined with a xenogeneic collagen matrix versus a connective tissue graft.

**Materials and methods:**

A total of 20 patients were randomized to receive one of two treatments: coronally advanced flap + xenogeneic collagen matrix (test group) and coronally advanced flap + connective tissue graft (control group). Patient-related outcomes measures and professional aesthetic assessment by root esthetic score were performed. A descriptive and analytical statistical analysis of the variables was performed.

**Results:**

At 12 months, the mean root coverage was 56.48% in the test group and 69.72% in the control group (*p* = 0.048), with a 35% and 40% complete root coverage in the xenogeneic collagen matrix and connective tissue graft, respectively. Test group presented less pain (3.65 vs. 5.2 VAS units) (*p* = 0.015) and less surgical time (45 vs. 49.15 min) (*p* = 0.004) than control group.

**Conclusion:**

The use of xenogeneic collagen matrix in RT2 recessions was effective for recession reduction to those obtained using autologous grafts; with the advantage that the duration of surgery and patient morbidity decreased. Therefore, xenogeneic collagen matrix in RT2 recessions could be an alternative to autologous grafts.

**Clinical relevance:**

The use of xenogeneic collagen matrix decreases the surgery time and patient morbidity but connective tissue graft results in significantly better mean root coverage and complete root coverage. Xenogeneic collagen matrix can be used in the treatment of RT2 gingival recessions.

**Study registration:**

NCT 03344315.

## Introduction

Gingival recessions are a very common pathology in the adult population [1, 2]. The ideal goal of recession treatment is to achieve complete root coverage (CRC) [3] and good aesthetic results of surrounding soft tissues [4].

In terms of the percentage of mean root coverage (MRC) and CRC, the treatment of choice is the combination of a subepithelial connective tissue graft (CTG) with a coronally advanced flap (CAF) [5–7]. The use of a CTG increases gingival thickness (GT) and achieves long-term stability [8] of the gingival margin [6, 9]. However, obtaining a CTG may cause postoperative complications [10, 11], thus leading to the development of different alternatives, such as membranes [12], biological agents [13], and allografts [14]. The objective of these therapies was to find a therapeutic alternative to the use of CTGs, thus allowing the treatment of multiple recessions in a single session, which can reduce surgery times, avoid the need for a second surgery, and improve the color and texture of the tissues [12–16]. This is especially important in patients with periodontitis and multiple recessions with interproximal attachment loss whose treatment is more complex [17].

The use of a xenogeneic collagen matrix (CMX) (Geistlich Mucograft®: Geistlich Pharma, AG, Wolhusen Switzerland) has been proposed as an alternative treatment for regeneration around teeth, obtaining promising results in comparison with CAF alone [6, 18, 19] in achieving greater root coverage and keratinized tissue. These studies have been performed in single and multiple RT1 [20] recessions with different follow-ups and surgical techniques such as CAF or Modified Advanced Coronal Tunneling Advanced (MCAT) [6, 18, 19]. While the percentage of MRC at 12 months with MCAT [21, 22] ranged from 53.2% [21] to 71% [22] the combination of CMX + CAF [8, 15, 23–26] showed better results in root coverage (76.28% [23] − 94.32% [26]).

RT2 [20] recessions are very prevalent in the adult population [6], but the CRC is not always predictable [27]. Although there are clinical studies in which CRC as well as high percentages of MRC have been achieved [28, 29], it is necessary to determine the predictability of treatment with autologous grafts as well as with possible alternative therapies [6, 30], such as the CMX.

Hence, this multicenter clinical trial aimed to compare the clinical efficacy of CMX (Geistlich Mucograft®: Geistlich Pharma, AG, Wolhusen Switzerland) versus a subepithelial CTG when combined to CAF [31] for the treatment of RT2 recessions in terms of percentage of MRC.

## Materials and methods

### Trial design

This was a randomized, double-blind, multicenter (2-center) clinical trial with a 12-month follow-up period. The study was registered in clinicaltrials.gov (NCT 03344315) and was conducted following the Consolidated Standards of Reporting Trials (CONSORT) guidelines [32]. The study was conducted following the principles of the Declaration of Helsinki (revised in 2013) and was approved by the Euskadi Ethics Committee (CEIC-E) (PI 20,161,008-PS) in February 2017.

The primary aim was to analyze the percentage of MRC associated with the treatment of recessions with CMX (test group) vs. CTG (control group), with the null hypothesis (H0) that both treatments are equally effective at 12 months. The secondary outcomes were the percentage of CRC, the reduction of recession (RECred) measured in mm, the gain in keratinized tissue width (KTW), the change in GT, patient-related outcome measures (PROMs) regarding postsurgical pain, satisfaction with the treatment and aesthetics perceived by the patient, and aesthetics by a blinded clinical monitor (MVF).

### Participants

#### Reference population

All participants were recruited from two private centers between March 2017 and May 2019. Patients received information about the treatment and the advantages and disadvantages of participating in this study. Informed consent was obtained from all participants before the start of the study.

Inclusion criteria were as follows: (a) patients ≥18 years old; (b) periodontally treated and healthy with at least one or more RT2 buccal gingival recessions in incisors, canines, and premolars, located in the same quadrant or sextant with a minimal depth of ≥ 2 mm; (c) full mouth plaque index had to be under 25% [33].

The exclusion criteria were as follows: (a) active periodontal disease; (b) subjects with severe systemic pathology who took or had taken medication that could interfere with the healing of periodontal tissues during the last 6 months.

#### Sample size calculation

For sample size calculation, the percentage of MRC was considered as the primary outcome to calculate the sample size. To provide a statistical power of 80%, an *α*-risk of 5%, and an SD = 14, as previously described in the literature [22], 18 patients would be necessary. To account for anticipated dropouts, an additional 10% was added, resulting in 20 patients.

#### Randomization

To determine the type of graft (CMX or CTG) to be used, the patients were randomized in blocks of two treatments using statistical software (IBM SPSS® Statistics 20.0) (AMGF). The allocation was kept hidden by a clinical monitor (AMGF) until the time of the intervention in opaque envelopes that were opened immediately after flap elevation.

#### Control of study bias

The clinical examiner (MVF) and the biostatistician (XMM) were blinded to the type of treatment (CMX or CTG). The reproducibility of the clinical examiner (MVF) was determined by evaluating 4 patients (presenting multiple RT2 recessions) not related to the study, at least twice, with a separation of at least 24 h. An intraclass correlation coefficient > 0.85 was considered acceptable.

### Intervention: Surgical procedure

All patients initially completed a plaque control program, including oral hygiene instructions [34] to correct habits related to the etiology as well as a presurgical prophylaxis. All the surgeries were performed by two experienced periodontists (LAAZ and ERGP), being the CAF [31] the technique chosen in all cases. The data related to the chair time, defined as the duration of the surgical procedure (in minutes) from the first incision to the last suture was also registered.

After preparing the recipient bed, a CMX (Geistlich Mucograft®: Geistlich Pharma, AG, Wolhusen Switzerland) (test group) or CTG (control group), which was obtained using the UPV/EHU technique [35], was implanted. Both the CMX and the CTG were sutured with absorbable sutures (Ethicon Vicryl® 5/0; Johnson & Johnson); the flap was sutured with Gore-Tex e-PTFE® 5/0 (W.L. Gore & Associates (UK), LTD, Scotland))

Postoperative care included a single presurgical dose of 2 ml of intramuscular betamethasone (Celestone® Cronodose®, Schering Plough S.A., Spain), amoxicillin 875 mg/clavulanic acid 125 mg (Augmentine®, GlaxoSmithKline S.A., Spain) every 8 h for 7 days, analgesics on demand (25 mg dexketoprofen) and rinses with 0.2% chlorhexidine digluconate (2 times a day for 3 weeks). Also, local cold applications for two days, a soft diet and no physical exercise during the first week after the surgery were advised. Sutures were removed from the palate at 7 days and from the recipient bed at 14 days respectively. At 21 days after surgery, daily brushing was resumed [34].

### Monitoring and data collection

At 7, 14 days, 4 weeks, 6, and 12 months patients were scheduled to attend follow-up appointments, which included a professional plaque removal with reinforcement of oral hygiene instructions.

#### Clinical parameters

At baseline, 6 and 12 months, an experienced, blinded, and previously calibrated examiner (MVF) recorded the following parameters using a standardized periodontal probe (PCP-11, Hu-Friedy, Mfg. Co. LLC, Chicago, USA); the measurements were rounded to the nearest 0.5 mm:

- Recession depth (REC depth): measured in the mid vestibular from the cemento-enamel junction (CEJ) to the deepest point of the pocket. As non-carious cervical lesions were not excluded, if the CEJ was not present or not detectable, it was determined using the technique described by Cairo and Pini-Prato [36] in 2010.
- Recession width (REC width): measured mesio-distally at the level of the CEJ.
- Keratinized tissue width (KTW): measured from the gingival margin to the mucogingival junction.
- Clinical attachment level (CAL), measured from the CEJ to the gingival margin to the deepest point of the pocket (PD + REC depth).
- Percentage of CRC (the number of treated recessions with REC depth = 0 mm): CRC × 100/number of recessions.
- Percentage of MRC (mean preoperative REC depth -mean postoperative REC depth/mean preoperative REC depth × 100) were calculated.
- GT: measured 3 mm apical from the center of the gingival margin (at baseline and 12 months) using a 25-gauge K endodontic file with a rubber stopper, measured perpendicular to the tooth axis under local anesthesia.

#### Patient-related outcomes measures

Regarding esthetics, a PROM was carried out using a visual analogue scale (VAS, ranging from 0 to10) in which patients were asked to score their satisfaction related to their clinical experience (procedure and postoperative pain) at 7 days and esthetic (0 = worst possible aesthetic result and 10 = excellent aesthetic result) at 12 months. Respecting the clinical experience (0 was considered the worst procedure experienced for the patient and 10 the best) and for postoperative pain (0 = no pain and 10 = extreme pain).

#### Professional esthetic evaluation

Besides the patient’s esthetic perception, the esthetics were also assessed from a professional point of view. Thus, one blinded and calibrated examiner (MVF) compared the photographs taken at baseline and 12 months postoperatively. The assessment was made using the Root Coverage Esthetic Score (RES) [37].

### Statistical analysis

All the obtained data were analyzed using statistical software (IBM SPSS® Statistics 20.0; IBM, Chicago, IL, USA), with the patient as the unit of analysis. Initially, the Shapiro–Wilk test was used to evaluate if the distribution was normal or not. First, descriptive statistics were performed, and means and standard deviations were provided for quantitative variables, and percentages were determined for categorical variables. Subsequently, in the analysis of statistical relationships, normality tests led to the use of nonparametric tests for both intragroup (Wilcoxon test of the ranges for related samples) and intergroup comparisons (Mann–Whitney *U*).

## Results

**Fig. 1:**
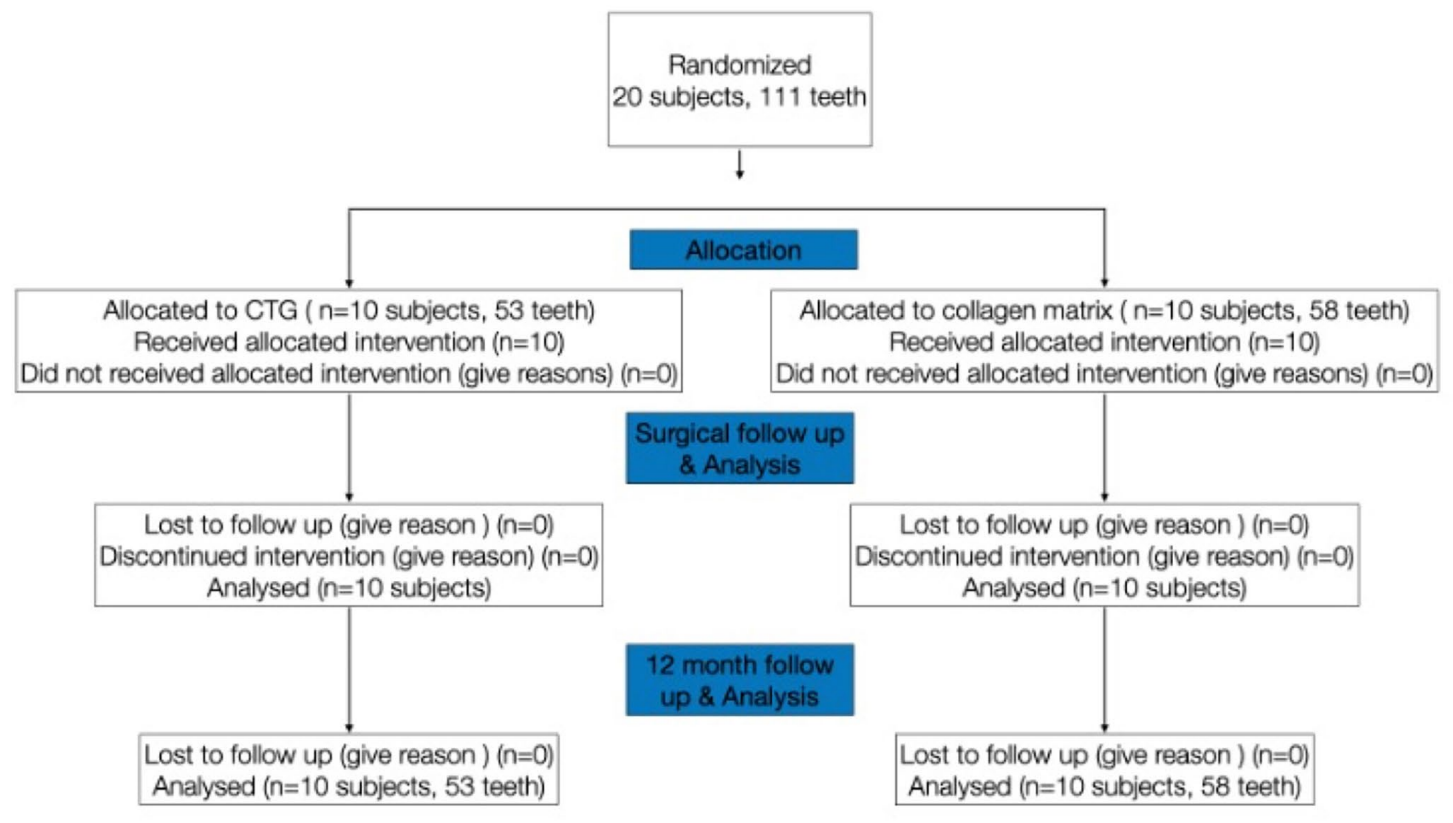
CONSORT diagram

The CONSORT diagram, which shows each group and the number of participants, as well as the number of drop-outs, is shown in Fig. 1.

### Study population

Twenty patients (13 women (65%)) with a mean age of 48.59 years (SD:10.32), of whom two were smokers (10%) participated in this study. The 96.39% of the participants had multiple recessions. A total of 111 RT2 recessions were treated (CMX: 58 vs. CTG: 53), among which 65 (CMX: 34 vs. CTG: 31) were in the mandible (58.5%). A total of 31 incisors (27.92%), 36 canines (32.43%), and 44 premolars (39.63%) were included. REC depth, REC width, KTW, GT, and CAL at baseline are shown in Table 1.

**Table 1:** Baseline characteristics of the study population

	Test (CMX) (*n* = 0)	Control (CTG) (*n* = 20)	*P* value	Total
Age (mean ± SD)	48.79 ± 10.35	48.39 ± 10.59	0.73	48.59 ± 10.32
Females n (%)	13 (65%)	13 (65%)	> 0.05	13 (65%)
Smokers n (%)	1 (5%)	1 (5%)		2 (10%)
REC per patient (mean ± SD) [range]	2.9 ± 1.12 [1–5]	2.65 ± 0.99 [1–4]	0.622	2.78 (1.05) [1–5]
REC maxilla n (%)	24 (41.38%)	22 (41.51%)	> 0.05	46 (41.44%)
REC mandible n (%)	34 (58.62%)	31 (58.49%)		65 (58.5%)
REC depth (mm) (mean ± SD) [range]	3.69 ± 1.03 [2.25–5.5]	3.67 ± 0.66 [2.25–4.75]	0.968	3.68 ± 0.85 [2.25–5.5]
REC width (mm) (mean ± SD) [range]	4.15 ± 0.73 [2.75–5.5]	3.91 ± 0.68 [2.5–5.5]	0.289	4.03 ± 0.71 [2.5–5.5]
CAL (mm) (mean ± SD) [range]	4.88 ± 1.12 [3.33–7]	4.95 ± 0.75 [3.25–6.5]	0.565	4.91 ± 0.95 [3.25–7]
KTW (mm) (mean ± SD) [range]	1.78 ± 1.24 [0–5]	1.77 ± 1.10 [0–4]	0.968	1.78 ± 1.24 [0–5]
GT (mm) (mean ± SD) [range]	1.09 ± 0.28 [0.5–2]	1.19 ± 0.29 [1–2]	0.365	1.14 ± 0.29 [0.5–2]

REC depth (Recession depth); REC width (Recession width); KTW (keratinized tissue width); CAL (Clinical attachment level); GT (Gingiva thickness); SD (standard deviation); mm (millimeters); CMX (xenogeneic collagen matrix); CTG (connective tissue graft)

### Clinical results

Clinical results at 6 months are summarized in Table 2. When analyzing the intergroup results, no significant differences were observed in any of the parameters analyzed. Clinical results at 12 months are reported in Tables 2 and 3, and 4; the changes between 6 and 12 months are shown in Table 3.

**Table 2:** Clinical results at baseline (T0), six (T1) and twelve (T2) months

	Grupo	T_0_	T1	T_2_	T0-T1	T0-T2	T1-T2	
		(baseline) (mean ± SD)	(6 months) (mean ± SD)	(12 months) (mean ± SD)	P intragroup	P intragroup	P intragroup	
RECdepth (mm)	Test (CMX) *n* = 20	3.69 ± 1.03	1.48 ± 0.73	1.55 ± 0.77	**< 0.001**	**< 0.001**	0.448	
	Control (CTG) *n* = 20	3.68 ± 0.66	1.18 ± 0.88	1.11 ± 0.78			0.905	
P (intergroup)		0.968	0.314	0.149	-	-	-	
RECwidth (mm)	Test (CMX) *n* = 20	4.15 ± 0.74	2.83 ± 1.02	2.9 ± 0.94	**< 0.001**	**< 0.001**	0.866	
	Control (CTG) *n* = 20	3.91 ± 0.69	2.38 ± 1.56	2.48 ± 1.58			0.767	
P (intergroup)		0.289	0.478	0.583	-	-	-	
KTW (mm)	Test (CMX) *n* = 20	1.79 ± 1.25	2.36 ± 2.47	1.96 ± 1.09	0.307	0.443	0.623	
	Control (CTG) *n* = 20	1.78 ± 1.11	2.47 ± 1.3	2.43 ± 1.17	**0.004**	**0.01**	0.752	
P (intergroup)		0.968	0.301	0.242	-		-	
CAL (mm)	Test (CMX) *n* = 20	4.88 ± 1.12	2.53 ± 0.78	2.64 ± 0.79	**< 0.001**	**< 0.001**	0.14	
	Control (CTG) *n* = 20	4.95 ± 0.76	2.35 ± 0.96	2.28 ± 0.87			0.284	
P (intergroup)		0.565	0.738	0.265	-	-	-	

REC depth (Recession depth); REC width (Recession width); KTW (keratinized tissue width); CAL (Clinical attachment level); SD (standard deviation); mm (millimeters); CMX (xenogeneic collagen matrix); CTG (connective tissue graft)

**Table 3:** Results of clinical changes at six (T1) and twelve (T2) months

Clinical parameter	Group	T1	T2	P intragroup
		(6 months)	(12 months)	
MRC (%)	Test (CMX) *n* = 20	59.60 ± 16.23	56.48 ± 19.68	0.167
	Control (CTG) *n* = 20	68.70 ± 23.53	69.72 ± 21.21	0.378
*P intergroup*		0.163	**0.048**	
CRC (%)	Test (CMX) *n* = 20	45	35	0.084
	Control (CTG) *n* = 20	40	40	0.086
*P intergroup*		0.64	0.14	
RECred (mm)	Test (CMX) *n* = 20	2.21 ± 0.90	2.14 ± 1.07	0.448
	Control (CTG) *n* = 20	2.50 ± 0.84	2.56 ± 0.86	0.905
*P intergroup*		0.277	0.181	
CAL gain (mm)	Test (CMX) *n* = 20	2.36 ± 0.97	2.64 ± 0.79	0.14
	Control (CTG) *n* = 20	2.60 ± 1.10	2.28 ± 0.87	0.284
*P intergroup*		0.512	0.221	
KTW gain (mm)	Test (CMX) *n* = 20	0.57 ± 2.08	0.18 ± 0.84	0.623
	Control (CTG) *n* = 20	0.69 ± 0.85	0.65 ± 0.92	0.752
*P intergroup*		0.174	0.114	
GT (mm)	Test (CMX) *n* = 20	-	0.46 ± 0.54	-
	Control (CTG) *n* = 20	-	0.78 ±0.47	-
*P intergroup*		-	**0.038**	

MRC (mean root coverage), CRC (complete root coverage), RECred (reduction in recession); KTW (keratinized tissue width); CAL (Clinical attachment level); GT (Gingival thickness); SD (standard deviation); % (percentage); mm (millimeters); CMX (xenogeneic collagen matrix); CTG (connective tissue graft)

**Table 4:** Clinical results in maxilla and mandible at 12 months of follow-up

Parameter T_2_	Location	CMX	CTG	p intergroup
MRC (%)	Maxilla (*n* = 9)	65.01 ± 16.83	83.34 ± 15.68	**0.015**
	Mandible (*n* = 11)	49.49 ± 19.74	58.58 ± 18.83	0.141
p intragroup		0.152	**0.007**	
CRC n (%)	Maxilla (*n* = 9)	55.56	77.78	**0.034**
	Mandible (*n* = 11)	27.28	9.09	0.72
p intragroup		0.412	**0.012**	
REC depth (mm) (mean ± SD)	Maxilla (*n* = 9)	2.61 ± 0.97	3.15 ± 0.73	0.297
	Mandible (*n* = 11)	1.76 ± 1.06	2.08 ± 0.67	0.217
p intragroup		0.08	**0.003**	
REC width (mm) (mean ± SD)	Maxilla (*n* = 9)	1.6 ± 0.99	2.5 ± 1.48	0.19
	Mandible (*n* = 11)	0.97 ± 0.89	0.57 ± 0.78	0.332
p intragroup		0.147	**0.001**	
KTW gain (mm) (mean ± SD)	Maxilla (*n* = 9)	0.16 ± 1.09	0.87 ± 1.07	0.185
	Mandible (*n* = 11)	0.19 ± 0.63	0.46 ± 0.77	0.401
p intragroup		0.943	0.33	
CAL gain (mm) (mean ± SD)	Maxilla (*n* = 9)	2.67 ± 0.97	3.47 ± 0.88	0.222
	Mandible (*n* = 11)	1.89 ± 1.07	2.01 ± 1.03	0.562
p intragroup		0.08	**0.004**	
GT gain (mm) (mean ± SD)	Maxilla (*n* = 9)	0.81 ± 0.55	0.97 ± 0.48	0.481
	Mandible (*n* = 11)	0.17 ± 0.31	0.64 ± 0.44	**0.034**
p intragroup		**0.004**	0.135	

MRC (mean root coverage), CRC (complete root coverage), RECred (reduction in recession); KTW (keratinized tissue width); CAL (Clinical attachment level); GT (Gingiva thickness); SD (standard deviation); % (percentage); mm (millimeters); CMX (xenogeneic collagen matrix); CTG (connective tissue graft)

The percentage of MRC was 56.48% on test and 69.72% on control sites at 12 months (*p* = 0.048). Between 6 and 12 months, the percentage of MRC decreased from 59.60 to 56.48% in the test group and an increased in the control group from 68.70 to 69.72%, respectively. The CRC was achieved in 7 patients (35%) in the test group and 8 patients (40%) in the control group Three patients presented with CRC for all treated recessions in the control group. At the recession level, the percentage of CRC decreased in the test group (from 15.5 to 12.1%), and in the control group, it increased from 18.9 to 22.6% from 6 to 12 months.

Both treatment groups showed significant post-surgical improvement in all clinical variables, except for the KTW in the test group. The GT increased after the surgical procedures: 0.46 mm (SD:0.54) in the test group and 0.78 mm (SD:0.47) in the control group (*p* = 0.038).

An analysis was performed by the treatment group (test group or control group) and the results are summarized in Table 4. Statistically significant differences were observed for all parameters (except KTW and GT) in the control group between the upper and lower jaws, with favorable results for the maxilla; in the test group, the differences between the maxilla and mandible were only significant for the increase in GT.

### Results of the surgical procedure and PROMs

No postoperative complications were observed after any surgery. The results are reported in Table 5. The mean surgical time was 45 min (SD:9.01), i.e., 41 min (SD:7.6) (CI 95% 37.41–44.59) in the test group and 49.15 min (SD:8.5) (CI 95% 45.15–53.15) in the control group; the differences between groups were significant (*p* = 0.004).

**Table 5:** Patient based surgical parameters and patient perception during the surgery

	Test (CMX)	Control (CTG)	intergroup p	
	*N* = 20 (mean ± SD)	*N* = 20 (mean ± SD)		
Surgical time (minutes)	41 ± 7.6	49.15 ± 8.5	**0.003**	
RES	6.74 ± 1.97	7.29 ± 2.24	0.309	
PROMs				
VAS postoperative pain	3.65 ± 2.08	5.2 ± 2.31	**0.015**	
VAS clinical experience	6.4 ± 2.21	5.35 ± 2.08	0.134	
VAS aesthetic	6.85 ± 1.5	7.15 ± 1.3	0.64	

Surgical time: duration of the surgical procedure (in minutes); RES (root coverage esthetic score), PROMs: clinical experience using a visual analog scale (VAS) (0 being the worst procedure and 10 the best); postoperative pain using a visual analog scale (VAS) (0 being the least pain and 10 being the maximum pain). Aesthetic result as assessed by the patient using a visual analog scale (VAS) (0 = worst possible aesthetic result and 10 = excellent aesthetic result) SD (standard deviation); mm (millimeters); CMX (xenogeneic collagen matrix); CTG (connective tissue graft)

Regarding the assessment of pain, less pain was reported by patients who received CMX, with a VAS = 3.65 (SD:2.08) (95% CI 2.67–4.63) vs. VAS = 5.2 (SD:2.31) (95% CI 4.12–6.28) (*p* = 0.015). Patients in the test group were more satisfied with the procedure in general, but the differences between groups were not significant.

The aesthetic evaluation score at 12 months was 6.85 (SD:1.5) in the test group and 7.15 (SD:1.3) in the control group These values were similar to the RES score in the test group (6.7 (SD:1.97)) and in the control group (7.28 (SD:2.24)).

**Fig. 2:**
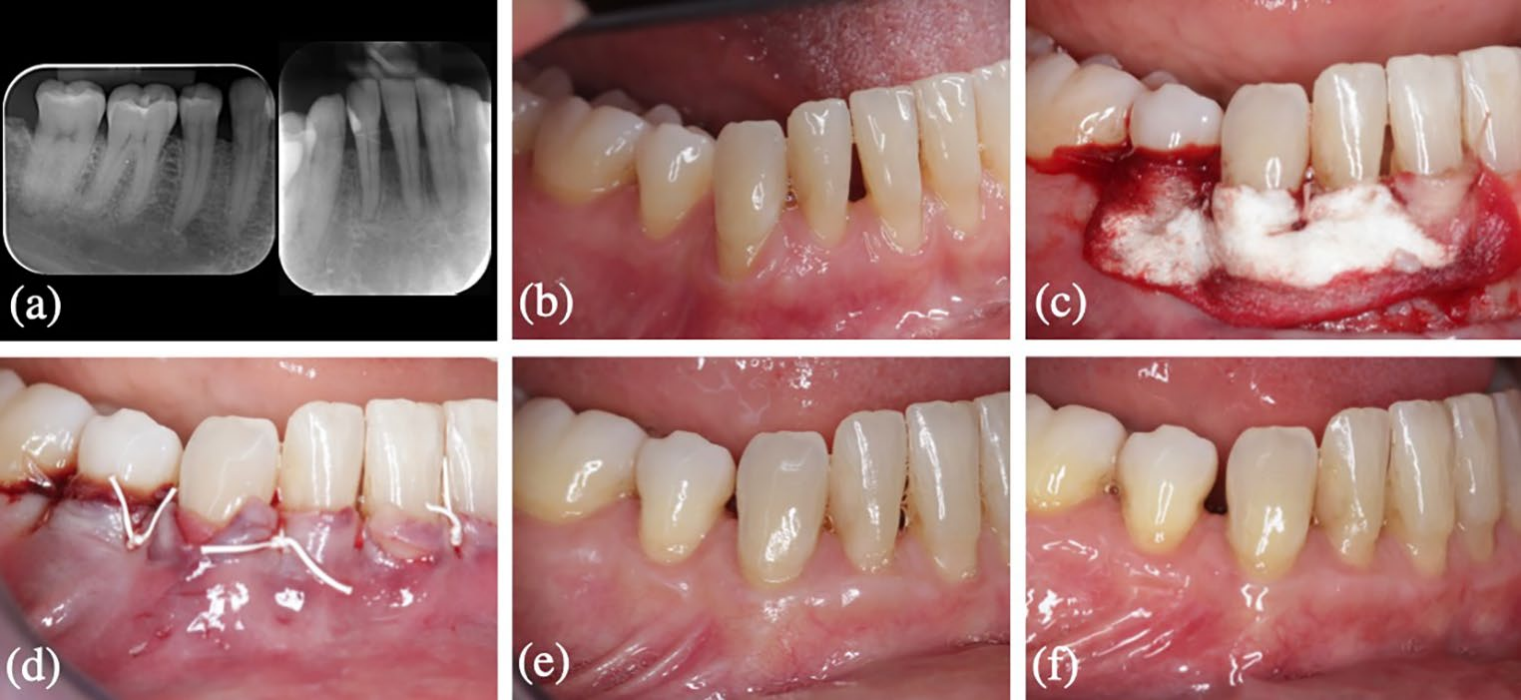
Surgical intervention in a patient allocated to the CAF + CMX group. (a)(b) clinical and radiographic features at baseline, (c) Intra-operative view: CMX, (d) Immediate postoperative view: coronally flap advancement and closure, (e)(f) Outcome at 12-months follow-up

**Fig. 3:**
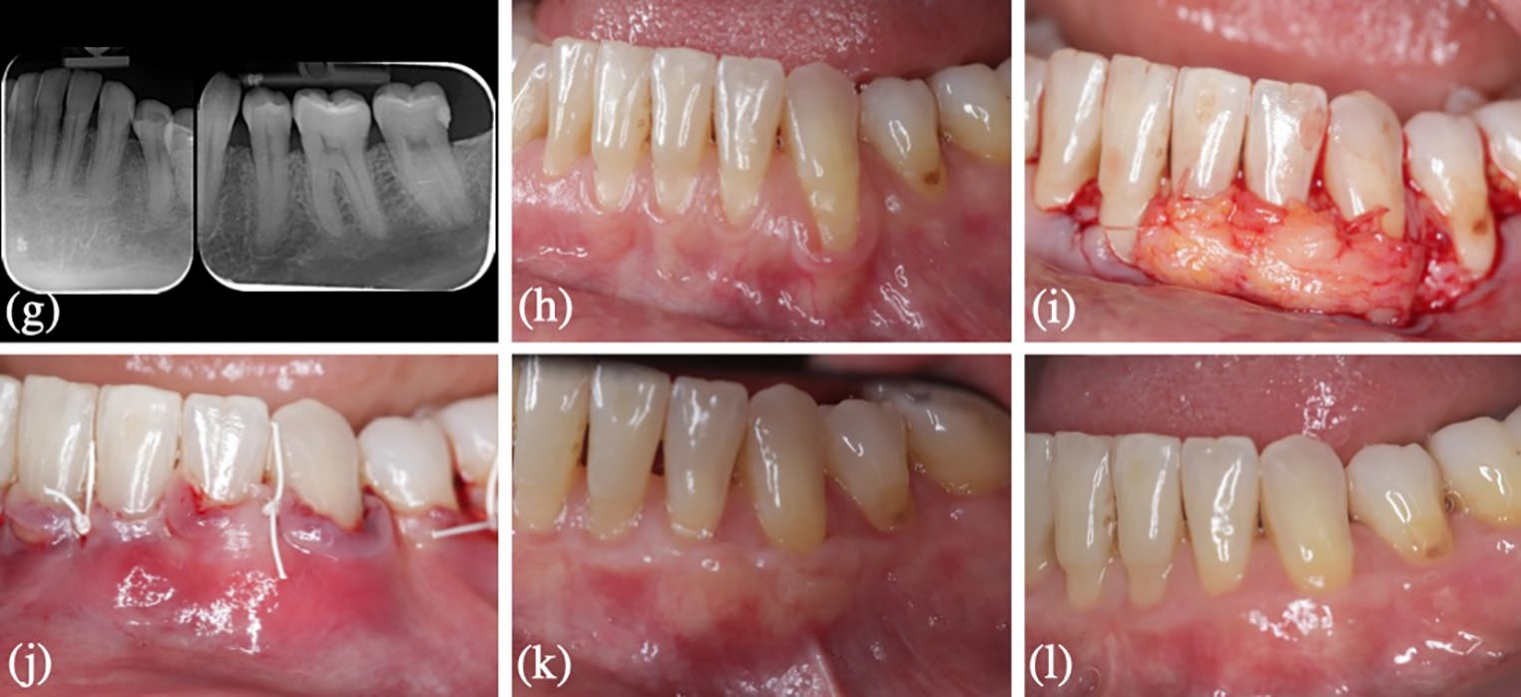
Surgical intervention in a patient allocated to the CAF + CTG group: (g)(h) clinical and radiographic features at baseline, (i) Intra-operative view: CTG (j) Immediate postoperative view: coronally flap advancement and closure (k)(l) Outcome at 12-months follow-up

Clinical and radiographic characteristics and surgical procedures of one patient from test group and control group at baseline and 12 months are shown in Figs. 2 and 3.

## Discussion

To our knowledge, there is no double-blind randomized clinical trial in the literature that compares the use of a soft tissue substitute such as the CMX vs. the CTG for the treatment of RT2 recessions in combination with CAF, thus making it difficult to compare our results with recent evidence [6, 27, 30].

The results of this study indicated that the percentage of MRC was lower in the test group than in the control group (CMX: 56.48% vs. CTG: 69.72%) (*p* = 0.048) and that the percentage of CRC was lower in the test group than in the control group (35% vs. 40%) after 12 months; therefore, hypothesis (H0) is rejected.

Our results partially coincide with a recent meta-analysis of Miller classes I and II recessions [18, 38], in which lower MRC were observed with CMX compared with CTG, with similar CRC in both study groups.

However, the percentage of MRC (CMX:56.48% vs. CTG: 69.72%) (*p* = 0.048) and the percentage of CRC (CMX:35% vs. CTG: 40%) results for both treatment groups were within the ranges reported in previous studies of RT2 recessions with CAF + CTG at 12 months (54.8-77.57%) [39–41]. Only, Fernández-Jiménez et al. [40], 2023, evaluated the CRC at 12 months in multiple recessions with results (50%) that were slightly superior to those herein (40%) where the KTW at baseline was higher.

The evidence regarding CMX is limited to RT1 recessions where different treatments have been compared: (a) CAF + CTG vs. CAF + CM [8, 15, 26] (b) CAF vs. CAF + CM [23–25, 42, 43]; (c) Modified Coronally Advanced Tunnel (MCAT) + CTG vs. MCAT + CM [22, 23]. The percentage of MRC with CMX at 12 months ranged from 76.2% [23] to 94.32% [24]. Our results were lower (56.48%) than the current evidence, probably due to the type of recessions treated in this clinical study (RT2) where attachment loss was present. Also, only three studies until now have treated multiple recessions [8, 24, 25] with CAF + CMX, and CRC was evaluated in five studies previously [8, 24–26, 42] and it ranged from 36% [42] to 72% [24]. Although these results were superior to those obtained in our study (35%), they are not comparable due to the type of recessions treated, where the RECred as well as KTW gain should be considered a treatment success in the treatment of gingival recessions associated with loss of interproximal attachment [44, 45].

In Chambrone’s 2010 meta-analysis [46], for the treatment of RT1 recessions, in studies with more than 10 patients, at 6 months, the CTG yielded very heterogeneous results, with percentages of MRC (64.5 − 97.3%) and percentages of CRC (10 − 96.1%) values that coincide with a previous review [3]. Therefore, our MRC results (CMX: 56.48% vs. CTG: 69.72%) for RT2 recessions are encouraging. However, CRC (CMX: 35% vs. CTG: 40%) is far from the results for recessions without insertion loss, which was expected based on evidence in the literature [6].

A recent CRC meta-analysis of 37 publications about these types of recessions [27] found wide CRC ranges (32.87 − 63.82%). Achieving CRC for these types of recessions is considered a challenge because of interproximal attachment loss [47], which implies an increase in the avascular surface [26, 48]. Our results are consistent with those previously reported [49] of Miller class III recessions treated with modified tunnel technique (TTM) in combination with CTG, where differences between the MRC (83%) and the CRC (40%) were reported. The differences with our results in MRC could be due to the surgical technique [39] and/or the characteristics of the sample (our recessions were wider and had a lower KTW).

Advantages of using alternative materials include the reduction in intraoperative time and patient morbidity [8], findings that were confirmed in this RCT. A reduction in surgical time of 8 min was observed in our study, less than that obtained in a recent study [50], where RT1 recessions were treated (15 min).

Patient satisfaction was generally higher in the test group, which also presented lower morbidity (*p* = 0.015); these findings confirm previous results [8], in which the recovery time of patients was shorter (1.8 days) in the CMX group, thus favoring a better assessment of the treatment received.

Another objective of these procedures is to increase keratinized tissue [18], which is key in recessions with fine phenotypes and in maintaining the GM in the long term [51, 52]. It has been reported that KTW gains are higher with CTG than with CMX, but without being significant [18], which coincides with our results at 6 months, but not at 12 months, where a greater decrease in KTW was observed in the CMX group. The evidence for increased GT with CMX vs. CTG is limited [25]; in this study, at 12 months, there was a trend for increased GT with the CTG (0.32 mm, *p* = 0.056), coinciding with a difference of 0.23 mm in favor of CTG in the study by Cardaropoli et al. [26]. In the CMX group, the thickness gain was greater in the upper maxilla (*p* = 0.004), unlike in the control group, where the differences between the maxilla and mandible were not significant.

The main indication for the treatment of recessions is aesthetic demand [53]. There is no consensus on which approach yields more aesthetic results concerning CRC or the similarity of treatment tissue with adjacent tissue [4, 45]. In our study, the aesthetic assessment by the patients was high and similar in both groups (CMX: 6.85 (SD:1.5) vs. CTG: 7.15 (SD:1.3)), a finding that coincides with previously reported results [24], where no differences were observed between groups. In contrast, a recent study obtained a more natural texture and contour with CMX than with a CTG [54]. When the professional assessment was performed the RES was similar between groups (CMX: 6.70 (SD:1.97) vs. CTG: 7.28 (SD:2.24)) and comparable to results reported in the literature [23], i.e., RES of 7.85 (SD:2.42) for CMX and 7.34 (SD:2.90) for CTG. Therefore, both treatment modalities achieved satisfactory aesthetic results for both professionals and patients.

Although this RCT shows new and relevant information on the treatment of RT2 recessions, it is not exempt from limitations. First, there was the lack of assessment of the flap thickness margin in surgery or the presence of detectable step or CEJ whose importance has been evidenced in the recent 2018 classification. Also, the soft tissue outcomes were assessed at 12 months, so long-term observations are necessary to confirm these results. However, this is the first double-blinded RCT in RT2 recessions that compares a CMX and autogenous grafts, including both maxillae (mandible:58.5%), and multiple recessions (96.39%).

## Conclusions

Within the limitations of this clinical study, although MRC was greater with CTG we conclude that CMX is a treatment option for these challenging RT2 recessions. The surgical time and patient morbidity were reduced in the test group. Therefore, CMX can be considered an alternative to autologous grafts.

It is necessary to perform more RCTs in patients with periodontitis due to the high prevalence of multiple recessions in these patients, which require large grafts that entail greater postoperative morbidity.

## Data Availability

No datasets were generated or analysed during the current study.

## References

[1] Heasman PA, Ritchie M, Asuni A, Gavillet E, Simonsen JL, Nyvad B (2017). Gingival recession and root caries in the ageing population: a critical evaluation of treatments. J Clin Periodontol.

[2] Romandini M, Soldini MC, Montero E, Sanz M (2020). Epidemiology of mid-buccal gingival recessions in NHANES according to the 2018 World workshop classification system. J Clin Periodontol.

[3] Cairo F, Pagliaro U, Nieri M (2008). Treatment of gingival recession with coronally advanced flap procedures: a systematic review. J Clin Periodontol.

[4] Kerner S, Sarfati A, Katsahian S, Jaumet V, Micheau C, Mora F (2009). Qualitative Cosmetic evaluation after Root-Coverage procedures. J Periodontol.

[5] Buti J, Baccini M, Nieri M, La Marca M, Pini-Prato GP (2013). Bayesian network meta-analysis of root coverage procedures: ranking efficacy and identification of best treatment. J Clin Periodontol.

[6] Chambrone L, Salinas Ortega MA, Sukekava F, Rotundo R, Kalemaj Z, Buti J (2018). Root coverage procedures for treating localised and multiple recession-type defects. Cochrane Database Syst Reviews.

[7] Chambrone L, Tatakis DN (2015). Periodontal soft tissue root coverage procedures: a systematic review from the AAP Regeneration Workshop. J Periodontol.

[8] Tonetti MS, Cortellini P, Pellegrini G, Nieri M, Bonaccini D, Allegri M (2018). Xenogenic collagen matrix or autologous connective tissue graft as adjunct to coronally advanced flaps for coverage of multiple adjacent gingival recession: randomized trial assessing non-inferiority in root coverage and superiority in oral health-related. J Clin Periodontol.

[9] Tavelli L, Barootchi S, Cairo F, Rasperini G, Shedden K, Wang HL (2019). The Effect of Time on Root Coverage outcomes: A Network Meta-analysis. J Dent Res.

[10] Griffin TJ, Cheung WS, Zavras AI, Damoulis PD (2006). Postoperative complications following gingival augmentation procedures. J Periodontol.

[11] Wessel JR, Tatakis DN (2008). Patient outcomes following subepithelial connective tissue graft and free gingival graft procedures. J Periodontol.

[12] Zucchelli G, Clauser C, De Sanctis M, Calandriello M (1998). Mucogingival Versus guided tissue regeneration procedures in the treatment of deep recession type defects. J Periodontol.

[13] McGuire MK, Nunn M (2003). Evaluation of human recession defects treated with coronally advanced flaps and either enamel matrix derivative or connective tissue. Part 1: comparison of clinical parameters. J Periodontol.

[14] Aichelmann-Reidy ME, Yukna RA, Evans GH, Nasr HF, Mayer ET (2001). Clinical evaluation of Acellular Allograft Dermis for the treatment of human gingival recession. J Periodontol.

[15] McGuire MK, Scheyer ET (2010). Xenogeneic collagen matrix with coronally advanced flap compared to connective tissue with coronally advanced flap for the treatment of dehiscence-type recession defects. J Periodontol.

[16] Wilson TG, Mcguire MK, Nunn ME (2005). Evaluation of the Safety and Efficacy of Periodontal Applications of a living tissue-Engineered Human fibroblast- derived dermal substitute. II. Comparison to the Subepithelial Connective tissue graft: a randomized controlled feasibility study. J Periodontol.

[17] Graziani F, Gennai S, Roldan S, Discepoli N, Buti J, Madianos P (2014). Efficacy of periodontal plastic procedures in the treatment of multiple gingival recessions. J Clin Periodontol.

[18] Alsarhan MA, Al Jasser R, Tarish MA, Alhuzaimi AI, Alzoman H (2019). Xenogeneic collagen matrix versus connective tissue graft for the treatment of multiple gingival recessions: a systematic review and meta-analysis. Clin Exp Dent Res.

[19] Atieh MA, Alsabeeha N, Tawse-Smith A, Payne AGT (2016). Xenogeneic collagen matrix for periodontal plastic surgery procedures: a systematic review and meta-analysis. J Periodontal Res.

[20] Cairo F, Nieri M, Cincinelli S, Mervelt J, Pagliaro U (2011). The interproximal clinical attachment level to classify gingival recessions and predict root coverage outcomes: an explorative and reliability study. J Clin Periodontol.

[21] Pietruska M, Skurska A, Podlewski Ł, Milewski R, Pietruski J (2019). Clinical evaluation of Miller class I and II recessions treatment with the use of modified coronally advanced tunnel technique with either collagen matrix or subepithelial connective tissue graft: a randomized clinical study. J Clin Periodontol.

[22] Aroca S, Molnar B, Windisch P, Gera I, Salvi GE, Nikolidakis D (2013). Treatment of multiple adjacent Miller class I and II gingival recessions with a modified coronally advanced tunnel (MCAT) technique and a collagen matrix or palatal connective tissue graft: a randomized, controlled clinical trial. J Clin Periodontol.

[23] Stefanini M, Jepsen K, De Sanctis M, Baldini N, Greven B, Heinz B (2016). Patient-reported outcomes and aesthetic evaluation of root coverage procedures: a 12-month follow-up of a randomized controlled clinical trial. J Clin Periodontol.

[24] Cardaropoli D, Tamagnone L, Roffredo A, Gaveglio L (2014). Coronally advanced flap with and without a xenogenic collagen matrix in the treatment of multiple recessions: a randomized controlled clinical study. Int J Periodontics Restor Dent.

[25] Rotundo R, Genzano L, Patel D, D’Aiuto F, Nieri M (2019). Adjunctive benefit of a xenogenic collagen matrix associated with coronally advanced flap for the treatment of multiple gingival recessions: a superiority, assessor-blind, randomized clinical trial. J Clin Periodontol.

[26] Cardaropoli D, Tamagnone L, Roffredo A, Gaveglio L (2012). Treatment of gingival recession defects using coronally advanced flap with a porcine collagen matrix compared to coronally advanced flap with connective tissue graft: a randomized controlled clinical trial. J Periodontol.

[27] Fernández-Jiménez A, García-De-La-Fuente AM, Estefanía-Fresco R, Marichalar-Mendia X, Aguirre-Urizar JM, Aguirre-Zorzano LA (2021). Complete root coverage in the treatment of Miller class III or RT2 gingival recessions: a systematic review and meta-analysis. BMC Oral Health.

[28] Cairo F, Cortellini P, Tonetti M, Nieri M, Mervelt J, Cincinelli S (2012). Coronally advanced flap with and without connective tissue graft for the treatment of single maxillary gingival recession with loss of inter-dental attachment. A randomized controlled clinical trial. J Clin Periodontol.

[29] Cairo F, Cortellini P, Tonetti M, Nieri M, Mervelt J, Pagavino G (2015). Stability of root coverage outcomes at single maxillary gingival recession with loss of interdental attachment: 3-year extension results from a randomized, controlled, clinical trial. J Clin Periodontol.

[30] Tatakis DN, Chambrone L, Allen EP, Langer B, McGuire MK, Richardson CR (2015). Periodontal soft tissue root coverage procedures: a consensus report from the AAP regeneration workshop. J Periodontol.

[31] Zucchelli G, De Sanctis M (2000). Treatment of multiple recession-type defects in patients with esthetic demands. J Periodontol.

[32] Schulz KF, Altman DG, Moher D, Group C (2010). CONSORT 2010 statement: updated guidelines for reporting parallel group randomised trials. BMJ.

[33] O’Leary TJ, Drake RB, Naylor JE (1972). The plaque control record. J Periodontol.

[34] Stillman PR (1932). A philosophy of the treatment of periodontal disease. Dent Digest.

[35] Aguirre-Zorzano L, García-De-La-Fuente A, Estefanía-Fresco R, Marichalar-Mendia X (2017). Complications of harvesting a connective tissue graft from the palate. A retrospective study and description of a new technique. J Clin Exp Dent.

[36] Cairo F, Pini-Prato GP (2010). A technique to identify and reconstruct the cementoenamel junction level using combined periodontal and restorative treatment of gingival recession. A prospective clinical study. Int J Periodontics Restor Dent.

[37] Cairo F, Rotundo R, Miller PD, Pini Prato GP (2009). Root Coverage Esthetic score: a system to evaluate the esthetic outcome of the treatment of Gingival recession through evaluation of clinical cases. J Periodontol.

[38] Miller PD (1985). A classification of marginal tissue recession. Int J Periodontics Restor Dent.

[39] Henriques PS, Pelegrine AA, Nogueira AA, Borghi MM (2010). Application of subepithelial connective tissue graft with or without enamel matrix derivative for root coverage: a split-mouth randomized study. J Oral Sci.

[40] Fernández-Jiménez A, Estefanía-Fresco R, García-De-La-Fuente AM, Marichalar-Mendia X, Aguirre-Urizar JM, Aguirre-Zorzano LA (2023). Comparative study of the modified VISTA technique (m-VISTA) versus the coronally advanced flap (CAF) in the treatment of multiple Miller class III/RT2 recessions: a randomized clinical trial. Clin Oral Investig.

[41] Mercado F, Hamlet S, Ivanovski S (2019). Subepithelial connective tissue graft with or without enamel matrix derivative for the treatment of multiple class III-IV recessions in lower anterior teeth: a 3-year randomized clinical trial. J Periodontol.

[42] Jepsen K, Jepsen S, Zucchelli G, Stefanini M, de Sanctis M, Baldini N (2013). Treatment of gingival recession defects with a coronally advanced flap and a xenogeneic collagen matrix: a multicenter randomized clinical trial. J Clin Periodontol.

[43] Moreira ARO, Santamaria MP, Silvério KG, Casati MZ, Nociti Junior FH, Sculean A (2016). Coronally advanced flap with or without porcine collagen matrix for root coverage: a randomized clinical trial. Clin Oral Investig.

[44] Miller PD (1987). Root coverage with the free gingival graft. Factors associated with incomplete coverage. J Periodontol.

[45] Rotundo R, Nieri M, Mori M, Clauser C, Prato GP (2008). Aesthetic perception after root coverage procedure. J Clin Periodontol.

[46] Chambrone L, Sukekava F, Araújo MG, Pustiglioni FE, Chambrone LA, Lima LA (2010). Root-coverage procedures for the treatment of localized recession-type defects: a Cochrane systematic review. J Periodontol.

[47] Saletta D, Pini Prato G, Pagliaro U, Baldi C, Mauri M, Nieri M (2001). Coronally advanced flap procedure: is the interdental papilla a prognostic factor for root coverage?. J Periodontol.

[48] Ozcelik O, Seydaoglu G, Haytac MC (2015). Prediction of root coverage for single recessions in anterior teeth: a 6-month study. J Clin Periodontol.

[49] Aroca S, Keglevich T, Nikolidakis D, Gera I, Nagy K, Azzi R (2010). Treatment of class III multiple gingival recessions: a randomized-clinical trial. J Clin Periodontol.

[50] Tonetti MS, Cortellini P, Bonaccini D, Deng K, Cairo F, Allegri M (2021). Autologous connective tissue graft or xenogenic collagen matrix with coronally advanced flaps for coverage of multiple adjacent gingival recession. 36-month follow‐up of a randomized multicentre trial. J Clin Periodontol.

[51] Agudio G, Chambrone L, Pini Prato G (2017). Biologic remodeling of Periodontal dimensions of areas treated with Gingival Augmentation Procedure: a 25-Year Follow-Up Observation. J Periodontol.

[52] Pini-Prato GP, Cairo F, Nieri M, Franceschi D, Rotundo R, Cortellini P (2010). Coronally advanced flap versus connective tissue graft in the treatment of multiple gingival recessions: a split-mouth study with a 5-year follow-up. J Clin Periodontol.

[53] Zaher C-A, Hachem J, Puhan MA, Mombelli A (2005). Interest in periodontology and preferences for treatment of localized gingival recessions. A survey among Swiss dentists. J Clin Periodontol.

[54] Pelekos G, Lu JZ, Ho DKL, Graziani F, Cairo F, Cortellini P (2019). Aesthetic assessment after root coverage of multiple adjacent recessions with coronally advanced flap with adjunctive collagen matrix or connective tissue graft: randomized clinical trial. J Clin Periodontol.

